# Age moderation of the association between negative subsequent memory effects and episodic memory performance

**DOI:** 10.1016/j.nbas.2021.100021

**Published:** 2021-09-13

**Authors:** Patrick J. Pruitt, Lingfei Tang, Jessica M. Hayes, Noa Ofen, Jessica S. Damoiseaux

**Affiliations:** a Institute of Gerontology, Wayne State University, 87 E. Ferry St., Detroit, MI 48202, United States; b Department of Psychology, Wayne State University, 5057 Woodward Ave. 7th Floor Suite 7908, Detroit, MI 48201, United States

**Keywords:** Memory formation, Lifespan, Functional MRI, Cognitive development, Subsequent memory

## Abstract

Negative subsequent memory effects in functional MRI studies of memory formation have been linked to individual differences in memory performance, yet the effect of age on this association is currently unclear. To provide insight into the brain systems related to memory across the lifespan, we examined functional neuroimaging data acquired during episodic memory formation and behavioral performance from a memory recognition task in a sample of 109 participants, including three developmental age groups (8–12, 13–17, 18–25 year-olds) and one additional group of older adults (55–85 year-olds). Young adults showed the highest memory performance and strongest negative subsequent memory effects, while older adults showed reduced negative subsequent memory effects relative to young adults. Across the sample, negative subsequent memory effects were associated with better memory performance, and there was a significant interaction between negative subsequent memory effects and memory performance by age group. Posthoc analyses revealed that this moderation effect was driven by a stronger association between negative subsequent memory effects and memory performance in young adults than children, and that neither children nor older adults showed a significant association. These findings suggest that negative subsequent memory effects may differentially support memory performance across a lifespan trajectory characterized by developmental maturation and support further investigation of this effect in aging.

## Introduction

Episodic memory is a core cognitive process that encompasses memory for personal experiences, which are tied to specific times and places. This ability to draw on previous experience enriches our quality of life and its impairment proves to be distressing. Behavioral evidence demonstrates that the capacity for episodic memory greatly improves in typical development from childhood to early adulthood [1], and investigations into the neural mechanisms of such development have largely focused on individual regions, such as the medial temporal lobe and prefrontal cortex. Previous studies generally show increased activation in the PFC supporting better memory performance but found more nuanced effects in in the medial temporal lobe [2], [3], [4]. Parallel to findings in development, episodic memory declines later in life, although longitudinal investigations have found that decline is more moderate and begins later in adulthood[5], [6], [7] than is suggested by results from cross-sectional approaches [6], [8]. As in development, the role of the medial temporal lobe in age-related differences or changes in episodic memory has garnered much attention [9], [10], [11] with larger hippocampal volume and reduced hippocampal atrophy generally predicting reduced episodic memory decline. Episodic memory is also susceptible to insult in certain forms of age-related cognitive impairment [12], with studies showing an overactivation in the PFC and reduced activation in the medial temporal lobe in impaired samples (see [13] for *meta*-analysis). While these region-specific studies have been informative, more systematic analyses considering the whole lifespan are lacking.

In examining the development and aging of episodic memory, the subsequent memory paradigm is frequently utilized [14], [15]. In this paradigm, participants encode a series of items and are later asked to discriminate between previously studied items and new items. With this paradigm adapted to fMRI, subsequent memory effects can be identified by contrasting the neural response to items that are subsequently remembered and those subsequently forgotten. Two kinds of subsequent memory effects can be identified, the “positive subsequent memory effect” (positive SME) and the “negative subsequent memory effect” (negative SME). The positive SME refers to brain regions which demonstrate higher response during study items that were subsequently remembered than those subsequently forgotten. Positive SME was commonly found in medial temporal lobe, fusiform gyrus, and prefrontal cortex [16]. On the other hand, several regions demonstrate the opposite effect, that is, higher response during subsequently forgotten items than remembered [16], termed the negative (or reversed) SME. Interestingly, the negative SME appears to result not from lower activation to subsequently remembered items relative to subsequently forgotten items, but rather greater *deactivation* to subsequently remembered items than subsequently forgotten items [17]. This effect is commonly found for regions in the default mode network [18], where stronger deactivations are observed when participants engage in external-oriented cognitive tasks [19]. The discovery of negative SME suggests that an additional cluster of regions may be functionally complementary to regions showing positive SME, possibly regulating internal-oriented mental processes that are unrelated to the task [16]. While most research in development and aging has focused on specific regions that generally show positive SME, the role negative SME plays is less often discussed. Therefore, exploration of the negative SME has the potential to provide a deeper and complementary understanding of the neural processes which support episodic memory formation and how it relates to lifespan development.

Recently, a few studies have investigated the relevance of the negative SME in the context of memory development. Chai et al. [20] characterized negative SME in the default mode network (DMN) in children and young adults ages 8 to 24. They identified a significantly higher magnitude of memory-related deactivation in young adults, such that higher magnitude of deactivation to subsequently remembered items relative to subsequently forgotten items were found in multiple nodes of the DMN in young adults, but not in children. In another study, Tang et al. [21] focused on the SME in the PFC in children ages 8 to 25. They found age-related increase in positive SME in inferior frontal gyrus, but age-related increase in negative SME in superior frontal gyrus. Conversely, studies examining the subsequent memory effect in older adults have found reduced magnitude of negative SME compared to younger adults [22], [23], such that older adults showed the least deactivation to subsequently remembered items relative to subsequently forgotten items. Taken together, these findings suggest that negative SME may develop over adolescence and deteriorate from early adulthood to late adulthood, coinciding with the trajectory of change for episodic memory performance.

Given the similarity in the lifespan trajectory of negative SME and behavioral measures of memory, it is plausible that understanding the linkage between these two may provide additional information on behavioral measures. Several previous studies have explored the link between negative SME and memory performance in development and aging. For example, Tang et al. [21] found in a sample of 83 participants including children and young adults an overall association between stronger negative SME and better memory performance, such that age-related increase in the level of negative SME mediates the developmental improvements in memory performance. These findings mirror effects found in samples spanning the adult lifespan, where participants who perform better on the memory paradigm also show stronger negative SME than low performers [22], [24], [25], [26], [27], [28], [29]. Notably, several studies [21], [22], [29] found that only negative, and not positive, SME was associated with memory performance. While this relationship has been found in developmental, adult, and aging samples independently, it is unclear whether the strength of this association is consistent across the lifespan. Therefore, given the lifespan trajectory of episodic memory capacity, we were interested in characterizing negative SME across the lifespan using the same task and to directly test whether the association between negative SME and memory performance differ as a function of age.

We investigated the effect of age group on the association between negative SME and memory performance in a sample of participants ranging in age from middle childhood to late-life. Participants completed a subsequent memory paradigm, with fMRI acquisition during memory encoding followed by a postscan recognition test. We predicted better performance and larger negative SME in young adults compared to children and older adults. Based on prior literature demonstrating an association between negative SME and memory performance [22], [24], [25], [26], [27], [28], [29], we predicted such an association in our sample.

Employing identical stimuli, similar experimental design and analyses protocols we thus addressed our study objective and tested the null hypothesis that the association of negative SME with memory performance does not differ between age groups. This null hypothesis would suggest that the relevance of regions demonstrating negative SME is consistent, across age, in explaining individual variability in memory performance. That is, from middle childhood to late adulthood, regions demonstrating a negative SME similarly support memory performance. The alternative hypothesis, that the association between negative SME and memory performance does differ between age groups, would suggest that the relevance of these regions in supporting episodic memory is different across age groups. That is, these regions may support memory performance in some age groups and not others.

## Methods

### Participants

We recruited 145 healthy participants from the Metro Detroit community, including younger participants ranging in age from 8 to 25 years (n = 101, 51 females, age 15.13 ± 4.97 years, mean ± standard deviation) and older adult participants ranging in age from 55 to 85 years (n = 44, 35 females, age 70.52 ± 7.60 years). Younger and older participants were recruited in separate studies which were developed in parallel, aiming to align study design parameters between the two. The main experimental task that serves as the basis of this study was first developed for testing with the younger participants and then adapted for testing with older participants. Testing of older and younger participants was conducted within an overlapping time period. Several methodological differences exist in the overall testing of the two cohorts and in the specific adaptation of the experimental paradigm. All analyses were carefully planned to overcome any modifications in experimental design and to optimize generalization across cohorts and validity of this unique lifespan study. A subset of participants in the present sample were also included in the samples of previously published work; that is, a subset of the older adults helped compose the sample in J.M. Hayes et al [30], while a subset of the children, adolescents and younger adults were included in the sample in Tang et al. [21]. See Supplementary Methods for more detailed description of the overlap across these samples. Detailed methods for data collection in each sample have been described in those articles; summaries of shared and distinct procedures for each group are provided below. All participants were right-handed as assessed using the Edinburgh Handedness Inventory [31], with no history of psychiatric or neurological disorders, and were free of MRI contraindications. Participants in the older adult group had scores ≥ 25 on the Mini-Mental State Examination [32], which is considered within the cognitively normal range [33]. Furthermore, all older adult participants performed in the cognitively normal range as determined by either clinical assessment or performance on Wechsler Memory Scale IV indices of no less than 1.5 standard deviations below the normative mean [34]for two or more indices. Older adult participants were additionally screened for current use of psychotropic medications, uncontrolled medical conditions, brain injury and radiation or chemotherapy for cancer treatment, which served as additional exclusion criteria. All participants ages 18 and older provided informed consent, while for participants younger than 18 parental consent was obtained and participants provided written or oral assent. Studies were approved by the Wayne State University IRB.

Data for one older adult was excluded for task non-compliance (miss rate > 93%). We handled missing data with listwise deletion, therefore two participants, one child and one adolescent, were excluded as they did not complete IQ testing. We excluded an additional 33 participants for neuroimaging data quality concerns. Such concerns included excessive motion (>20% outlier volumes, volume-to-volume motion spike > 1.5 voxel width), fMRI signal dropout, and an unbalanced number of trials between conditions (ratio > 4) (Supplementary Table 1). Following these exclusions, a total of 109 datasets were included (Table 1).

**Table 1:** **Participant demographics (n = 109) by age group.** Gender distribution and IQ differ across age groups, with older adults having a higher proportion of women than children, adolescents, and young adults, and lower IQ than children and young adults.

Participant characteristics	Children	Adolescents	Young Adults	Older Adults	Test-statistic	p
Sample Size	23	28	29	29		
Age	9.96 ± 1.36	15.29 ± 1.36	21.03 ± 2.15	70.52 ± 7.60		
Gender (F/M)	11/12	15/13	16/13	24/5	8.469↑	0.037
IQ	109.65 ± 13.72	105.04 ± 14.22	110.66 ± 12.25	99.14 ± 11.26	4.692↑↑	0.004
Average framewise displacement	0.25 ± 0.11	0.17 ± 0.05	0.15 ± 0.05	0.24 ± 0.11	9.020↑↑	< 0.001
% outlier volumes	6.15 ± 4.56	4.59 ± 3.33	3.49 ± 2.12	4.75 ± 3,78	2.495↑↑	0.064

↑ chi-squared

↑↑ F

To examine the effect of age we grouped participants into four age groups in our analyses. Three age groups were defined from the sample of younger participants: children (age 8–12 years), adolescents (age 13–17 years), and young adults (age 18–25 years). These cut-offs were chosen to roughly align with general stages of puberty, with children in early puberty, adolescents in puberty, and young adults in post-puberty. Due to sample size the older adults were kept as a single age group (age 55–85 years). We characterized age using groups, rather than as a continuous variable, due to the gap between the upper limit of our younger adults (25 years) and the lower limit of our older adults (55 years). Participant demographics across groups are presented in Table 1. Gender distribution significantly differed across age groups, *χ^2^* = 8.47, *p* = 0.037, Cramer’s V = 0.237, as the older adult sample had a larger proportion of female participants than the children. We assessed participant IQ in the children, adolescents, and young adults using the Kaufman Brief Intelligence Test version 2 [35]; (scores were not available for one child and one adolescent) and in the older adults using the Wechsler Abbreviated Scale of Intelligence II [36]. These measures reflect a composite of crystallized and fluid intelligence. Group analysis showed that IQ significantly differed across age groups, *F*(3,105) = 4.07, *p* = 0.004, as the older adults had lower IQ scores than the children and young adults. Gender was included as a covariate in all statistical models, and IQ was included as a covariate in models for which memory performance was the dependent variable.

### Subsequent memory task

#### Stimuli

Participants were presented with indoor and outdoor scenes from a stimulus set used in prior studies [20], [21], [37], [38]. The stimulus set included a total of 240 scenes. Specific stimuli were equally likely to be included for study or recognition across participants.

#### Paradigm

Participants were presented with scenes while lying in the MRI scanner. Participants were instructed to make an indoor/outdoor judgment for each scene using a 2-button response box placed in their right hand. In addition, participants were instructed to try their best to memorize the scenes for a subsequent recognition memory test. The scanned study phase was followed by a recognition test conducted outside of the scanner, approximately 30 min after the end of the study phase. At recognition test, participants made a two-part response to each scene. First, participants made judgments on whether the scene presented was “old” or “new”, depending on whether they had seen the picture during the encoding phase. Subsequently, participants indicated their confidence in the “old/new” judgment by making a “sure/not sure” confidence decision. All participants were asked to reflect on how sure they were regarding their “old/new” decision although children, adolescents, and young adults were also specifically instructed to use “sure” in response for a picture they decided was previously seen, if they “remember anything specific about it” or “not sure” if it “just looks familiar”. The older adult participants were asked after each image to decide if they were sure or unsure of their “old/new” decision.

All participants studied scenes while in the scanner and given the recognition test outside the scanner though some specific details were different between the samples. All differences in procedure were accounted for in analyses to generate equivalence for comparison across the full age range across the two samples (age 8–85 years). Children, adolescents, and young adults studied 120 scenes in three consecutive runs (3 sets of 40 scenes per run, each set with 20 indoor and 20 outdoor scenes). Each scene was shown for 3 s followed by an intertrial interval of 0.5 s + 0–8 s (jittered, increments of 2 s). The assignment of 40-scene sets for study or recognition was counterbalanced across participants, with each participant allocated 1 of 6 pseudorandom list orders. Eighty additional scenes (2 sets of 40 scenes, each set with 20 indoor and 20 outdoor scenes) were used as foils in a subsequent recognition test. Older adults studied 80 scenes from the same stimulus set used in the sample of younger participants in one run (40 indoor and 40 outdoor). Each scene was shown for 3.4 s followed by an intertrial interval of 1 s + 0–11 s (jittered, increments of 2.2 s). Sixty additional scenes were randomly selected from the stimuli pool to be used as foils in the subsequent recognition test. Put succinctly, participants in the younger groups studied 120 scenes during encoding and 80 foils were added during recognition, while participants in the older adult group studied 80 scenes during encoding with 60 foils added during recognition. All participants practiced the task prior to entering the scanner and were reminded of the instructions.

Our paradigm utilizes intentional encoding rather than incidental encoding; the latter is often used to minimize differences in spontaneous encoding strategies between older and younger adults. We chose to employ intentional encoding because adults often anticipate a recognition task to follow while children may not. By acknowledging the recognition task, children are brought to a level playing field in expecting a later recognition task.

Scenes were presented via an Avotec Silent Vision (SV-6011) projection system and were viewed by participants through a mirror mounted on the head coil. The task was programmed and presenting using PsychToolbox (3.0.14, [39] via MATLAB (R2016b; MathWorks, Natick, MA) for the children, adolescents, and young adults and using E-Prime 2.0 (Psychology Software Tools, Pittsburgh, PA) for the older adults.

### Behavioral analysis

Encoding task accuracy was calculated as the proportion of correct indoor/outdoor responses during encoding. Encoding task accuracy was used as general means to assess individual attention during encoding. Trials that did not receive a correct indoor/outdoor judgment were removed from calculation of behavioral subsequent memory performance, and were not included in subsequent fMRI contrasts of interest.

Recognition responses were classified based on recognition accuracy and confidence in recognition judgement. Accuracy was characterized by whether participants correctly identified scenes studied during encoding as old and foils as new. Confidence ratings (high or low) were indicated by participants for each response at recognition. Trials in which studied scenes were subsequently recognized with high confidence were labelled high-confidence hit trials (Hit-HC), whereas trials in which scenes were subsequently recognized with low confidence were labelled low-confidence hit trials (Hit-LC). Trials in which studied scenes were later classified as ‘new’ were labelled as miss trials (Miss) regardless of the confidence rating. Foils that were falsely endorsed as ‘old’ with high confidence were labelled high-confidence false alarms (FA-HC). Recognition accuracy was determined as the proportion of Hit-HC (out of the total number of studied scenes correctly classified as indoor/outdoor) – the proportion of FA-HC (out of the total number of foils). Low-confidence responses were not included in the calculation of recognition accuracy as such responses are less likely to indicate reliable discriminability between remembered items and foils [22]. Recognition accuracy for low-confidence responses were all close to chance (0) for the sample overall (0.01 ± 0.06) and for each age group (Supplementary Table 2), supporting the idea that low-confidence responses may reflect a process closer to guessing than successful memory formation. Behavioral analyses were computed using SPSS (v25, IBM). Effects of age group on behavioral measures were calculated using one-way ANOVA, for which significant effects were further investigated using Tukey’s Honestly Significant Difference.

**Table 2:** **Task accuracy and subsequent memory performance by age group.** Both task behavioral measures differed across age groups. Encoding task accuracy was the proportion of correct indoor/outdoor judgments during encoding. Recognition accuracy was calculated by subtracting the proportion of high-confidence false alarms from the proportion of high-confidence hits. Children’s task accuracy (indoor/outdoor judgments of stimuli) was lower than that of older adults. Recognition accuracy of children and older adults was lower than that of young adults.

Behavioral measure	Overall	Children	Adolescents	Young Adults	Older Adults	F	p
Encoding task accuracy *(n = 91)*	0.96 ± 0.05	0.94 ± 0.04	0.96 ± 0.05	0.95 ± 0.07	0.99 ± 0.03	3.15	0.029
Recognition accuracy (*n = 109*)	0.27 ± 0.15	0.20 ± 0.12	0.28 ± 0.14	0.37 ± 0.16	0.23 ± 0.11	8.10	<0.001

### MRI data acquisition

All scans were conducted at the Wayne State University MR Research Facility at Harper University Hospital (Detroit, MI), with a 3-T Siemens Magnetom Verio scanner using a 32-channel Head Matrix coil. T1-weighted whole-brain anatomy images were acquired using a magnetization-prepared rapid gradient-echo sequence. Children, adolescents, and young adults: 192 sagittal slices, repetition time (TR) = 2200 ms, echo time (TE) = 4.26 ms, flip angle (FA) = 9°, field of view (FOV) = 256 mm, 192 × 256 voxels, and voxel size = 1 mm × 0.5 mm × 1 mm. Older adults: 176 slices, TR = 1680 ms, TE = 3.51 ms, FA = 9°, FOV = 256 mm, voxel size = 0.7 mm × 0.7 mm × 1.3 mm).

Functional images were acquired using a T2*-weighted gradient-echo sequence (Children, adolescents, and young adults: 30 slices parallel to the AC–PC plane, TR = 2000 ms, TE = 30 ms, FA = 90°, voxel size = 3.1 mm × 3.1 mm × 4 mm; older adults: 37 slices parallel to the AC-PC plane, TR = 2200 ms, TE = 30 ms, FA = 80°, FOV = 220 mm, voxel size = 2.8 mm × 2.8 mm × 2.8 mm). For children, adolescents, and young adults, the encoding task was completed in three consecutive functional runs of 118 volumes, encoding 40 scenes in each, or 120 scenes in total. For older adults, the task was completed in one functional run of 276 volumes, encoding 80 scenes in total.

### Imaging analysis

The major consideration in developing our analytic approach was to assure that we can combine data collected with slightly different parameters in the two samples. We note that partial data from each of the samples were included in our prior publications [21], [30] and below we describe how the analytic approach used here differed from that described in those prior publications. To allow us to combine data from the two samples we broadly adopted the analytic approach and implementation as utilized in Tang et al. [21] and applied it to data from both samples with a few key adjustments. Most notably, in the present work we added a threshold for maximum framewise displacement to reduce the potential detrimental effect of motion artifacts and utilized SPM12 rather than SPM8. We note that although we followed a different processing pipeline for the data previously used in J.M. Hayes et al. [30] the pipeline utilized in that study was conceptually similar to the one described here, only performed in FSL. Therefore we did not expect this change to be a source of inconsistency in findings across studies.

#### Preprocessing

We used FSL_motion_outliers (FSLv5.0.8, FMRIB’s Software Library, https://fsl.fmrib.ox.ac.uk/fsl/; [40]) to determine the maximum volume-to-volume framewise displacement for each run. Twenty-one participants with a maximum framewise displacement>1.5 times the voxel width (4.65 mm for children, adolescents and young adults, 4.12 mm for older adults) were excluded from analysis to prevent contamination of group results by motion spike-induced signal artifacts (12 children, 1 adolescent, 1 young adult, 7 older adults). In the remaining participants, there was an effect of age group on average framewise displacement, *F*(3,105) = 9.02, p < 0.001, *η^2^_P_* = 0.205, as children and older adults demonstrated greater displacement than adolescents and young adults (see Table 1). Average framewise displacement was therefore included as a covariate in the ANCOVA models for which a neuroimaging measure was the dependent variable.

Functional imaging data were then analyzed with the SPM12 package (v6906; Wellcome Department of Imaging Neuroscience, London, UK; https://www.fil.ion.ucl.ac.uk/spm/software/spm12/) running on MATLAB (R2016b). Images were motion-corrected, normalized to the Montreal Neurological Institute (MNI) template, and smoothed with an 8 mm full-width half-maximum Gaussian kernel. Additionally, we screened functional images using Artifact Detection Tools (http://www.nitrc.org/projects/artifact_detect/) to identify outlier volumes in pre-processed data. Specifically, an outlier volume was identified if (1) the global mean intensity of the volume was >3 SD from the mean volume intensity of the run, or (2) volume-to-volume difference of a composite motion parameter exceeded 1 mm. Two participants for whom > 20% of volumes were identified as outliers were excluded from analysis (1 child and 1 older adult). In the remaining participants, percent of outlier volumes did not differ between age groups, *F*(3,105) = 2.50, *p* = 0.064, *η^2^_P_* = 0.067. Following all exclusions, individual-level models were created for 109 participants.

#### Individual-level models

Individual-level general linear models (GLMs) included 3 task-related regressors for each run: high-confidence hits (Hit-HC), low-confidence hits (Hit-LC), and misses (Miss). Seven motion regressors (three translational and three rotational motion parameters, and one composite motion parameter as is the default output in ART) were also included per run. Individual-level GLMs also included an “error” regressor for trials in which the participant made an incorrect indoor/outdoor judgment or did not make a button response; therefore only trials that were given a correct encoding task response were included in group-level imaging analysis. Only these trials were included in task contrasts to better ensure that stimuli included in the analyses were appropriately attended to. For 18 participants the button presses did not register during encoding due to technical difficulties (2 children, 4 adolescents, 3 young adults, 9 older adults). These data were still included in imaging analyses, in line with prior protocols using these samples [21], [30], because encoding accuracy was high in all age groups, and we did not find evidence of a difference in recognition accuracy between those with (*M* = 0.28 ± 0.14) or without (*M* = 0.24 ± 0.16) encoding data (*T*(1 0 7) = 1.028, *p* = 0.306). Children, adolescents, and young adults completed three encoding runs and overall 120 scenes compared to a total of 80 encoded scenes by older adults. We therefore elected to include only the two runs with the lowest average framewise displacement in the individual-level models of the children, adolescents, and young adults. We made this choice, rather than using the first two runs, to reduce the group differences in motion and utilize more participant data. For example, several children had large motion spikes in either the first or second encoding run. If we opted to use only the first two runs, these participants would be excluded. By instead selecting the runs with the lowest motion, we can discard the run with the spike and use the other two runs. This approach brings the average framewise displacement of the children (the highest motion age group) in line with the other age groups, to the extent that average framewise displacement in children is not significantly higher than that in older adults (Table 1).

Each encoding event was modeled as an impulse function and convolved with a canonical model of the hemodynamic response function. Temporal derivatives were included in the GLM to account for any temporal shifts in response to the stimuli [41]. To minimize the influence of motion artifacts, we added one regressor per outlier volume into the GLM model (as identified by ART). Individual-level analyses were limited with a brain mask created by summing the normalized white and gray matter images generated from segmenting individual T1-weighted image using SPM12. Two contrasts were created for each participant: Hit-HC > Miss (positive SME) and Miss > Hit-HC (negative SME). The task contrasts were limited to those hits which received high confidence judgments (Hit-HC) as we are particularly interested in items which are successfully remembered.

#### Group analyses

Individual contrasts for positive and negative SME were submitted to separate group models. Group models included age group as a factor, and gender and average framewise displacement as covariates. In these voxelwise analyses, we corrected for multiple comparisons using family-wise error correction (*p* < 0.05 FWE). Clusters resulting from these analyses were saved as a binary mask, and individual contrast values were extracted using the positive and negative SME masks as regions of interest (REX; [42]), such that contrast values for each participant were averaged across all voxels in the mask. That is, we opted to average across all regions in the mask, therefore studying a large single region of interest rather than perform analyses on the individual regions. We chose this approach because we were interested more broadly in the set of regions showing negative subsequent memory effect rather than differences between individual regions. Additionally, we optimize statistical power using a single region of interest, rather than needing to correct for multiple tests across several individual regions of interest. Additionally, we included all regions showing positive or negative SME in the binary masks rather than attempting to characterize age-dependent vs age-invariant effects. Previous work characterizing age-dependent/invariant effects has primarily focused on differences between two or three age groups spanning early to late-life adulthood, which allows for straightforward interpretation of age-dependence or -invariance. Conversely, our lifespan sample spans two major periods of development, which we expect to show opposing age effects, complicating potential interpretation of age-dependence**.**

Effects of age group on positive and negative SME were calculated using ANCOVA models with gender and average framewise displacement as covariates. We investigated the overall association of SME with recognition accuracy using linear regression models that also included age group, gender and IQ. Subsequent memory contrast values were mean-centered. To determine whether age group moderated this association, we repeated the previous linear regression models with the addition of an interaction term of age group and mean-centered SME. A significant interaction term and change in *R^2^* were interpreted as evidence of moderation of the association between SME and recognition accuracy by age group. Reported brain region labels for group analyses were derived from the AAL atlas [43]. To account for multiple comparisons, alpha was adjusted by Bonferroni correction for the number of models run.

## Results

### Behavioral data

#### Task accuracy

Encoding task accuracy was very high with 96.1% ± 5.1% of the scenes correctly classified as indoor/outdoor during encoding. Encoding task accuracy, however, differed by age group, *F*(3,87) = 3.51, *p* = 0.029, with accuracy of children significantly lower than that of older adults (Table 2). We removed from subsequent memory analyses those scenes that participants did not accurately classify. By removing from analyses those scenes that were not correctly classified as indoor/outdoor during encoding we diminished age differences in attentional effects in encoding.

As mentioned above (Methods section 2.5.2), indoor/outdoor judgments were not recorded for a subset of participants due to technical issues (*n* = 18; 2 children, 4 adolescents, 3 young adults, 9 older adults), therefore the encoding task accuracy results presented here reflect data from the remaining 91 participants. Given the very high encoding accuracy in the rest of the sample, we elected to include all data from the 18 participants for whom encoding judgments were not recorded in subsequent behavioral and imaging analyses.

#### Subsequent memory performance

High confidence hit and false alarm rates significantly differed by age group (Hit-HC: *F*(3,105) = 4.11, *p* = 0.008; FA-HC: *F*(3,105) = 6.98, *p* < 0.001). Hit-HC rates were lower for children than young and older adults. FA-HC rates were higher for older adults than adolescents and young adults. Low-confidence hit and false alarm rates did not differ by age group (*p* > 0.1).

Recognition accuracy (proportion of Hit-HC – proportion FA-HC) across all participants was 0.27 ± 0.14. Recognition accuracy differed by age group, *F*(3,105) = 8.10, *p* < 0.001, with recognition accuracy in children and older adults significantly lower than that in young adults (Table 2, Supplementary Fig. 1).

**Fig. 1:**
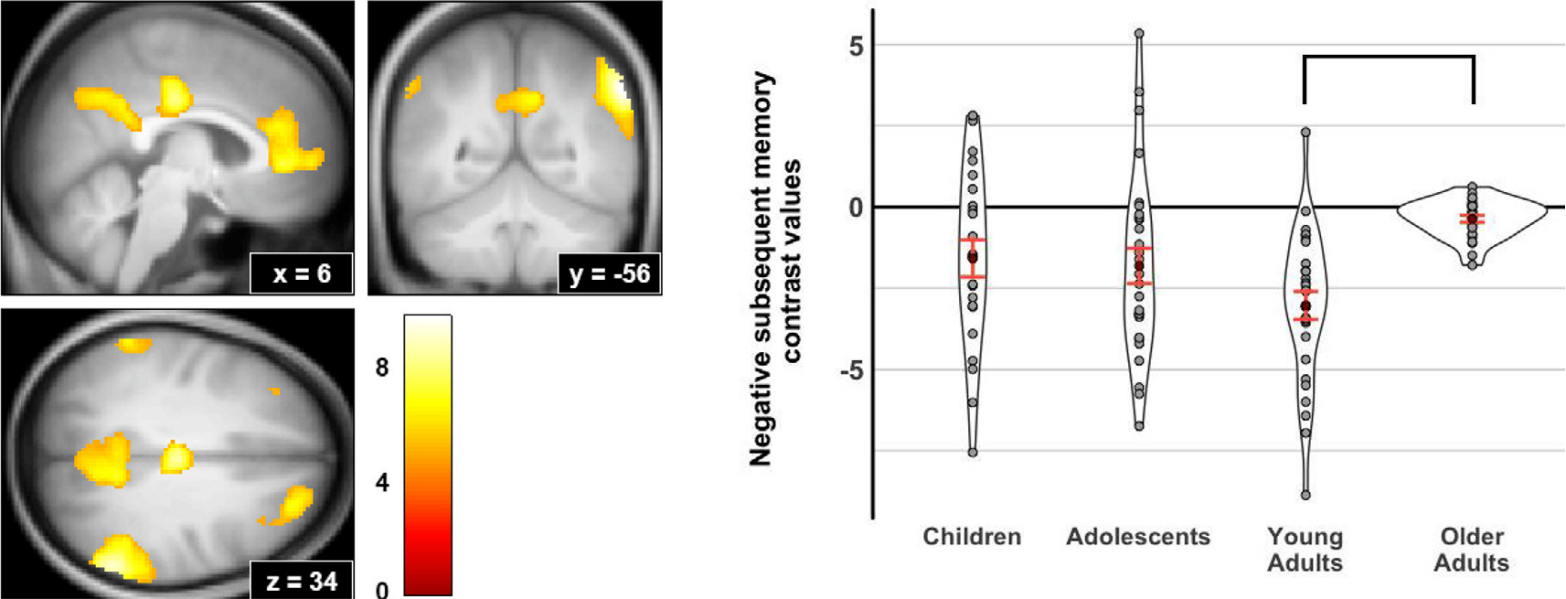
**Negative subsequent memory effect across and between age groups.***Left:* Negative SME contrast (high confidence hit > miss, negative values) shows significant clusters in medial prefrontal cortex/anterior cingulate, posterior cingulate/ precuneus, and bilateral inferior parietal cortex. We identified regions using a voxelwise significance threshold of *p* < 0.05 FWE-corrected. Significant clusters are displayed on the sample’s averaged T1 structural image; coordinates are provided in MNI space. *Right:* Violin plots demonstrating magnitude of negative SME is strongest in young adults, and comparatively reduced in older adults. There is a significant effect of age group on negative SME, *F*(3,103) = 5.47, *p* = 0.002, *η^2^_P_* = 0.14 . The dark red circles identify age group means, and error bars reflect ± 1 standard error. (For interpretation of the references to colour in this figure legend, the reader is referred to the web version of this article.)

We examined effects of age group and subsequent memory outcome (Hit-HC, Miss) in encoding judgment reaction time. Specifically, we tested whether the outcome effect in encoding judgment reaction time differed by age group (i.e. an interaction effect between age group and outcome). There was not a significant interaction effect between age group and outcome: *F*(3,87) = 2.51, *p* = 0.064. Put another way, the difference in encoding judgment reaction time between subsequent high-confidence hits and subsequent misses did not significantly differ by age group. Therefore, we did not include encoding judgment reaction time in our main analyses. See Supplementary Table 2 for additional information regarding outcome rates and encoding judgment reaction times by age group.

As noted in the Methods, the current implementation of the subsequent memory task was developed for the younger participants and later adapted for older adult participants. There are, therefore, some procedural differences described there. Of particular note, older participants completed one encoding run of 80 stimuli, while younger participants completed three encoding runs each with 40 stimuli, with the two lowest-motion runs used for analysis. Please refer to the Methods for a more complete description of methodological differences in the subsequent memory task for younger and older participants.

### Subsequent memory effect and age

Subsequent memory effects were assessed by contrasting activations for subsequently remembered items (Hit-HC) with activations for subsequently forgotten items (Miss). In this study we focus on *negative SME*—activation to subsequent Miss relatively above activation to subsequent Hit-HC (analyses of positive SME are reported in the Supplementary Materials). Negative SME was identified in regions including medial prefrontal cortex, posterior cingulate/precuneus, and bilateral inferior parietal cortex (Fig. 1, Supplementary Table 3). To test for an effect of age group on negative SME we extracted contrast values averaged across all voxels in these regions as a single region of interest per participant. (Exploratory analyses of *regional* negative SME are reported in the Supplementary Materials). There was a significant effect of age group on negative SME, *F*(3,103) = 5.47, *p* = 0.002, *η^2^_P_* = 0.14 (Fig. 1), controlling for gender and average framewise displacement. Posthoc pairwise comparisons of estimated marginal means revealed that negative SME was lower in older adults compared to young adults (*p* = 0.001); no other pairwise comparison reached statistical significance. See Supplementary Fig. 2 for statistics regarding pairwise age group comparisons, and Supplementary Table 4 for the results of the full model. The effects of age group on negative SME remained when co-varying recognition accuracy, in addition to gender and framewise displacement.

**Table 3:** **Pairwise group comparisons of association between negative subsequent memory effect and recognition accuracy.** Association between negative SME and recognition accuracy was significantly stronger in young adults compared to children. There were no other significant group differences.

Group comparison		t	p
Children vs	Adolescents	−1.653	0.102
	Young Adults	−3.114	0.002
	Older Adults	−0.178	0.859
Adolescents vs	Young Adults	−1.733	0.086
	Older Adults	0.328	0.743
Young Adults vs	Older Adults	0.854	0.395

**Fig. 2:**
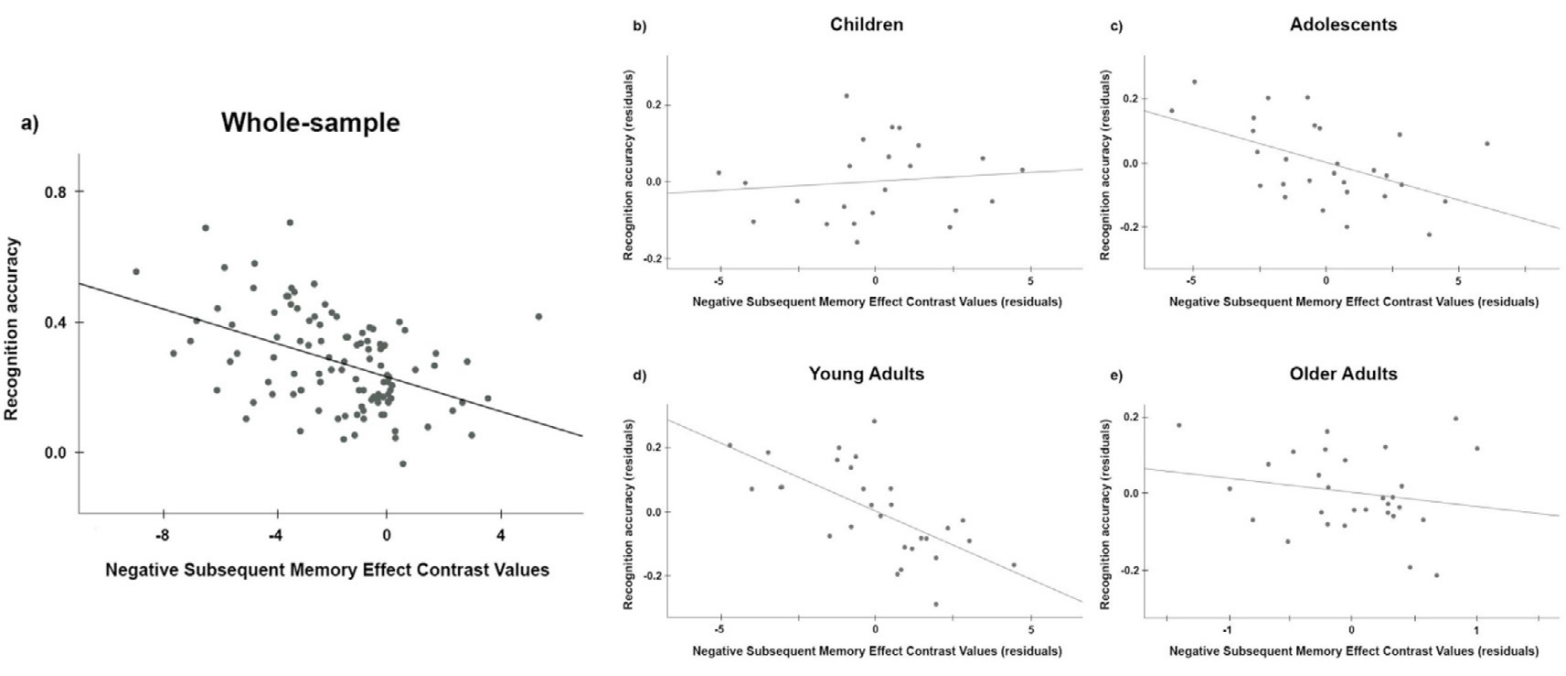
**Association between negative subsequent memory effect and recognition accuracy, across and within age groups.***a)* Scatterplot demonstrating that over the whole sample, negative SME was strongly associated with recognition accuracy, such that more negative subsequent memory effects are associated with better performance. Controlling for age group, gender, and IQ: *β* = -0.43, *t* = -5.14, *p* < 0.001. Age group moderated this association, *β* = -0.86, *t* = -3.73, *p* < 0.001, ΔR^2^ = 0.084. *b-e)* Partial regression plots demonstrate that, examining this association within age groups and after controlling for age (as integer), gender, and IQ, residualized negative SME was strongly associated with recognition accuracy in adolescents (*β* = -0.50) and young adults (*β* = -0.62), but not in children (*β* = 0.11) and older adults (*β* = -0.20).

### Subsequent memory effect and recognition accuracy

We were interested in whether negative SME was associated with behavioral memory performance in our sample, as this association has been found in previous studies. Our regression model predicting recognition accuracy included negative SME, age group, gender, and IQ. The overall regression model was significant: *F*(4,104) = 10.87, *p* < 0.001, adjusted *R^2^* = 0.27. Negative SME was significantly associated with recognition accuracy after controlling for age group, gender, and IQ: *β* = -0.43, *t* = -5.14, *p* < 0.001 (Fig. 2a), such that stronger negative SME was associated with better recognition accuracy. Results of the full model are reported in Supplementary Table 7 (Model 1).

Given that negative SME was associated with recognition accuracy across the whole sample, we investigated whether age group moderated this association by adding an age group by negative SME interaction term to the regression model. The interaction term was significant (*β* = -0.86, *t* = -3.73, *p* < 0.001) and its addition significantly improved the model (ΔR^2^ = 0.084). Full results of this analysis are provided in Supplementary Table 7 (Model 2).

We followed up with two sets of post hoc analyses to better interpret the moderation of the association between negative SME and recognition accuracy by age group. First, we used separate linear regression models for each age group to evaluate the association between negative SME and recognition accuracy, controlling for gender, IQ, and age in years. These within-group analyses revealed that negative SME was strongly associated with recognition accuracy in adolescents (*β* = -0.50, *t* = -2.88, *p* = 0.008) and young adults (*β* = -0.62, *t* = -4.27, *p* < 0.001), but not in children (*β* = 0.11, *t* = 0.49, *p* = 0.63) or older adults (*β* = -0.20, *t* = -0.97, *p* = 0.34) (Fig. 2b-e). Given that recognition accuracy and IQ covary in our sample, we repeated the post hoc analyses without IQ as a covariate and found the same pattern of results.

Second, we conducted pairwise group comparisons of the strength of the association between negative SME and recognition accuracy using the PROCESS macro for SPSS [44], [45]. To facilitate comparison between age groups, the moderator variable (age group) was coded as a nominal variable (multicategorical indicator). Threshold for statistical significance was set to 0.05, without correction for multiple comparisons per the recommendation of A.F. Hayes & Montoya [45]. These comparisons revealed that the strength of the association between negative SME and recognition accuracy was significantly stronger for young adults than for children (*t* = 3.114, *p* = 0.002). All other pairwise group comparisons were not statistically significant (Table 3).

## Discussion

In this study, we used a subsequent memory paradigm to investigate the neural correlates of memory formation across the lifespan. We focused our investigation on negative subsequent memory effects; such effects were previously documented separately in studies of memory development or aging. To demonstrate the functional relevance of negative SME across the lifespan we assessed the association between negative SME and memory performance and the moderation of this association by age in four age groups: children, adolescents, young adults and older adults. Memory performance differed by age group, with young adults performing better than children and older adults in line with prior work and the notion that episodic memory develops in early life and deteriorates in later life [20], [23], [25], [46]. As predicted, we observed robust negative SME in several regions consistent with previous reports [16]. The magnitude of negative SME differed by age and was reduced in older adults relative to young adults [22], [23]. Moreover, pursuant to our goal of assessing the functional role of negative SME we found that magnitude of negative SME across the sample was associated with recognition accuracy. In testing our study objective, we found that age group moderated the association between negative SME and recognition accuracy, driven by a significantly stronger association in young adults compared to children. Descriptive, within-group follow-up analyses found a strong association in adolescents and young adults but not in children or older adults. This moderated association provides further insight into differences in the negative SME across development, adulthood, and late-life: not only do performance and brain activity differ between groups but the association between them also differs, most notably across development.

Our particular pattern of age-differential findings is consistent with several previous reports. De Chastelaine and Rugg [29] found an association between negative SME and memory performance in younger adults, and Miller et al [24] who found an association between negative SME and memory performance across their adult lifespan sample. Amlien et al [47] examined encoding/retrieval flip, rather than subsequent memory effect, in their study of episodic memory across the lifespan. While encoding/retrieval flip is a distinct measure from subsequent memory effect, most notably in that it also accounts for brain response during encoding, both can broadly be considered as measures of activity modulation in response to memory processes. Our results are consistent with Amlien et al’s findings that activity modulation in response to memory processes differs across the lifespan, and that this modulation is associated with memory performance. Regarding older adults specifically, a significant association between negative SME and memory performance in this age group has been identified in some previous work [26], [27], [28], and has been absent in others [22], [23]. Consistent with the latter set of studies, but inconsistent with the former, the association was not significant in our older adult sample. The cause of this discrepancy is not immediately clear, although several points are worth consideration. First, the older adult group in the present sample includes participants across a 30 year age range (55–85 years), which is broader than the older adult age ranges in several similar previous studies[26], [28]. Evidence from previous literature demonstrates cross-sectional differences and longitudinal changes in both cognitive function (e.g. [6], [8] and neuroimaging measures (e.g. Giorgio et al. [48], [49], [50], [51] across this range. It may be that heterogeneity in our older adult sample driven by a wide age range may inhibit our ability to find the association reported in previous work. However, Mormino et al [27] did find a significant association in their older adult sample with an even broader age range (58–97 years), and de Chastelaine et al [26] did not find a significant association in their older adult sample with a relatively tight age range (63–76 years), suggesting that the lack of a significant association in our older adult sample cannot solely be attributed to the age range. Second, the sample size for our older adult group (n = 29) is smaller than several previous studies of this association in older adults. De Chastelaine et al [26] and Mormino et al [27] found a significant association in their older groups with sample sizes of 36 and 45 participants, respectively. However, Mattson et al [28] did find a significant association in their older adult sample with a smaller sample size (n = 25), and de Chastelaine et al [26] did not find a significant association in their older adult sample with a much larger sample size (n = 64). So while sample size is a consideration, we do not believe it accounts for the discrepancy between our finding and previous reports of a significant association in older adults. Third, previous reports of an association between negative SME and memory performance in older adults [26], [27], [28] have not controlled for IQ, which often correlates with memory performance. It may be the case that when accounting for IQ, the association between negative SME and memory performance would not be significant. That said, we do not find a significant association in our older adult sample even if IQ is not controlled for. Fourth, while we do not find a significant association between negative SME and memory performance in our older adults, the association in this group is not significantly weaker than any of the younger groups. That is, the significant association found in young adults, for example, is not significantly stronger than the association in older adults. Therefore, we must be cautious in our interpretation of the lack of an association in older adults, and the nature of this effect at the older end of the lifespan. A narrative has emerged in the literature that a significant association between negative SME and memory performance has been consistently demonstrated in older adults, however our results join several other studies in not finding such an association. However, given previous work that has found this association in older adults, further investigation is needed to more clearly characterize the impact of age on this association in later life.

The association between SME and overall memory performance was significant in adolescents and young adults. This finding is consistent with prior evidence pointing to the importance of negative SME in determining individual differences in memory [21], [29]. Negative SME could be indicating cognitive processes associated with effective re-allocation of cognitive resources including disengagement of task-irrelevant systems to facilitate engagement of task-relevant systems during successful compared to unsuccessful encoding. Consistent with this notion, the association of negative SME with overall memory performance in adolescents and young adults indicates that individuals who perform better, engage more efficient re-allocation of cognitive resources. In contrast, in both the children and older adults we did not observe a significant association between negative SME and overall memory performance. The lack of such association in children, given the overall strong negative SME in this group, indicates that merely having negative SME may not yet serve a functional role of determining individual differences in memory. This may be akin to an optimization of the brain regions where negative SME effects are observed across development, such that greater deactivation in these regions during encoding of subsequently remembered stimuli becomes directly related to better recognition accuracy with age. Collectively, the specificity of association between negative SME and memory performance in adolescents and young adults underscores the functional significance of negative SME and demonstrates clear differences in these effects across development.

Notably, regions in which negative SME were identified closely overlap with the regions of the default mode network, a set of regions which consistently deactivate during external cognitive processing and are thought to facilitate internally-focused processing [18]. Age-related optimization of negative SME for performance may be associated with age-related differences in maturation of this network. Indeed, evidence suggests nodes of the default mode network undergo increased integration throughout childhood. Children ages 7–9 show weak connectivity relative to young adults, particularly between medial prefrontal cortex and posterior cingulate cortex – two core network nodes[52], [53], but then show increased network integration from 10 to 13 [54] and into adulthood. This suggests that even within our youngest age group (8–13 years) default mode network maturation is taking place. In aging, there is reduced integration in healthy older adults relative to younger adults. Andrews-Hanna et al [55] found a negative association between age and functional connectivity between the medial prefrontal cortex and posterior cingulate cortex, with a steady decline in connectivity across their sample spanning ages 60–93 years. The declines in connectivity found in cross-sectional analyses show even greater disruption in older adults with cognitive impairment and dementia [56], [57], [58], in a longitudinal analysis, found that consistent declines in within-network default mode connectivity began around age 74, with a trend toward increased connectivity in older adults up to that age. This suggests that default mode connectivity trajectory in older adults may be nonlinear, even across the older adult age range used in the present study.

These interpretations come with two limitations. First, negative SME reflects only a subset of the default mode network. While the practical functional implications of this distinction between regions which deactivate during all encoding and regions which deactivate more during successful encoding are not currently clear [29], the presence of this distinction implies that negative subsequent memory results overlapping with the default mode network should be interpreted with caution. Second, empirical support for optimization of memory performance associated with system maturation and deterioration would require a longitudinal design. While the present work demonstrates age group *differences* in subsequent memory effect in a cross-sectional design, it does not demonstrate *changes* in the systems underlying subsequent memory effect. Further work is needed to investigate the link between memory performance and longitudinal development of subsystems of the default mode network across the lifespan.

In our regression models, we evaluated how several factors and covariates, including magnitude of negative SME, account for variability across individuals in a measure of memory performance. Noting that subsequent memory effect is, itself, a measure of successful memory performance, it is important to clarify the value in looking for associations between the two. Here, we must emphasize that the magnitude of subsequent memory effect is a within-subject measure, identifying brain regions for each individual which demonstrate differences in signal between successful (high confidence hit) and unsuccessful (miss) encoding. From this, we identify subsequent memory effect-associated brain regions across all age groups. However, the question remains of whether the magnitude of subsequent memory effect in these regions account for the variance in memory performance across individuals. For this, we conducted a between-subjects analysis: a regression model investigating the association between subsequent memory effect and recognition accuracy. As both are indicators of successful memory performance, one may expect that they should be associated. However, just because a participant has higher or lower magnitude of effects in specific brain regions during hit compared to miss does not necessarily imply they will have higher or lower frequency of hits compared to misses. It is this question that we have investigated across age groups, and our results suggest that the variance in recognition accuracy accounted for by this within-subject measure (negative SME) differs by age group in a between-subjects analysis. Put another way, we have identified a dissociation in which recognition accuracy and negative SME are associated in adolescents and young adults but not in children and older adults.

We acknowledge our approach has limitations which must be considered when interpreting the findings. First, some parameters for fMRI acquisition and task design in the children, adolescents, and young adults differ from the older adults (e.g. TR; acquired voxel size; stimulus duration). There were additional protocol differences as well: wording of instructions was not identical, and while the individual-level general linear models include an equal number of trials from all participants, these trials were collected across two shorter runs for children, adolescents, and young adults and one longer run for older adults. However, given expected age-group patterns of memory performance and overall neural task effects in expected regions, we believe direct comparisons between the developmental and older adult samples are warranted despite minor methodological differences across groups. Second, relatively few of our participants had enough low confidence hit (Hit-LC) trials for the use of that condition in task contrasts to be sufficiently powered. As a result, we were unable to delineate subsequent recollection (Hit-HC > Hit-LC) and subsequent familiarity (Hit-LC > Miss) effects, which are conflated in the Hit-HC > Miss contrast used in this study. Previous work (e.g. [28]) has suggested this may be an important distinction particularly in the context of age effects, as recollection appears more impaired in aging relative to familiarity [60]. Third, differences in BOLD response across groups may reflect differences in neural response but may also be attributed to age-related differences in vasculature and their effects on cerebral blood flow and the shape of the hemodynamic response function. We aimed to account for this potential variance by including temporal derivatives in the first level model [41], but the relative contributions of neural and vascular group differences to the reported results is unknown. Fourth, while we present findings from age groups across the lifespan, our sample does not include adults from ages 25–55, limiting our ability to determine where in adulthood the reported brain-behavior association begins to degrade. Inclusion of this age range in future studies will further clarify the moderating effect of age on brain-behavior associations. Fifth, the age-dependent associations described in the present study are based on cross-sectional, rather than longitudinal data. While this allows us to examine *differences* among age groups across the lifespan, we are not able to examine *change* within individuals as they age. Longitudinal data may show different patterns of aging than are found in cross-sectional data. The current results must be evaluated with this in mind, and replication of these effects in longitudinal data are needed to more strongly support the proposed maturation and decline of the association between negative SME and memory performance. Sixth, in this study we focus on memory sensitivity rather than attempting to assess age group differences in metamemory, or an individual’s knowledge of their own memory. Development of metamemory enables utilization of mnemonic strategies [59], meaning that individual or age group difference in metamemory may also support differences in the memory processes captured with fMRI. While examination of metamemory is beyond the scope of the current analysis, the extent to which age differences in metamemory may explain age differences the brain-behavior relationship is an intriguing question for future research. Seventh, while we believe our approach of averaging across all regions demonstrating a subsequent memory effect was appropriate for our study, it does sacrifice regional specificity. The mask included regions that are functionally distinct, such as the middle frontal gyrus and posterior regions commonly associated with the default mode network. Exploratory regional analyses are reported in Supplementary Material, however future studies that are sufficiently powered to correct for tests across multiple regions may provide more nuanced, region-specific insight into the effects investigated here. Eighth, our older adult age group features participants across an age range from 55 to 85 years. While similar studies have also used broad ranges for their older adult samples [24], [27], previously reported differences in both cognitive function [8,6] and neuroimaging measures [48], [49], [50], [51] across this range are not accounted for here. Individuals in a relatively broad range of older adulthood are included in a single age group in order to optimize statistical power. In addition to the inclusion of a mid-life sample, multiple groups of older adults with smaller age ranges and more targeted region-specific analyses may further clarify the nature of this moderation effect in adulthood.

In conclusion, our findings demonstrate that the association between negative SME (ostensibly reflecting a subset of the default mode network) and memory performance is different across developmental age groups, most strongly between children and young adults, suggesting developmental maturation of successful memory facilitation by the default mode network. While the association was not significant in older adults, neither was it significantly weaker than in younger age groups, thus supporting the need for further investigation into this effect in older adults using a larger sample and more finely-grained age-groupings. Such a follow-up investigation would help clarify whether the developmental effect demonstrated here is mirrored by age-related deterioration in late-life. That said, this work supports a role for negative SME in age-related differences in episodic memory across typical development and potentially across the lifespan.

## CRediT authorship contribution statement

**Patrick J. Pruitt:** Conceptualization, Formal analysis, Writing - original draft, Visualization. **Lingfei Tang:** Conceptualization, Formal analysis. **Jessica M. Hayes:** Investigation, Data curation. **Noa Ofen:** Conceptualization, Writing - review & editing. **Jessica S. Damoiseaux:** Conceptualization, Writing - review & editing, Supervision.

## Declaration of Competing Interest

The authors declare that they have no known competing financial interests or personal relationships that could have appeared to influence the work reported in this paper.
