# Supplementary material for: Age moderation of the association between negative subsequent memory effects and episodic memory performance
Source: Aging Brain. 2021 Sep 13;1:100021. doi: 10.1016/j.nbas.2021.100021 (PMC9997129; doi:10.1016/j.nbas.2021.100021)
Supplement: Supplementary data 1 [file mmc1.docx]

**Supplementary Materials**

*Methods 2.1: Participants*

Data from 20 children (87.0% of current age group sample), 21 adolescents (75.0%), and 27 young adults (93.1%) have been included in analyses reported on in a previous publication (Tang et al., 2018). In addition, data from 18 older adults (62.1% of current older adult sample) have been included in analyses reported on in a separate previous publication (J.M. Hayes et al., 2017). As a whole, in addition to this study being the first time where data used in these two prior publications is being combined, in the analyses presented here we added 21.1% new data sets (from 23 new participants not included in prior published work).

**Supplementary Table 1. Exclusions by age group.**

|  | **Overall** | **Children** | **Adolescents** | **Young Adults** | **Older Adults** |
| --- | --- | --- | --- | --- | --- |
| Recruited | 145 | 38 | 31 | 32 | 44 |
| IQ score not available | 2 | 1 | 1 | 0 | 0 |
| Miss rate > 93% | 1 | 0 | 0 | 0 | 1 |
| Motion spike > 1.5 voxel width | 21 | 12 | 1 | 1 | 7 |
| >20% outlier volumes | 2 | 1 | 0 | 0 | 1 |
| Hit-HC/Miss ratio > 4 | 9 | 1 | 1 | 2 | 5 |
| fMRI signal dropout | 1 | 0 | 0 | 0 | 1 |
| Analysis sample | 109 | 23 | 28 | 29 | 29 |

*Results 3.1.1: Task accuracy*

**Supplementary Table 2. Recognition outcome rate and encoding judgment reaction time by age group.** The rates of high-confidence hits and high-confidence false alarms differed across age groups. Children’s average high-confidence hit rate were lower than those of young adults and older adults. Older adults’ average high confidence false alarm rate was higher than those of adolescents and young adults. Recognition accuracy for low-confidence responses was close to chance for all age groups, suggesting these responses may reflect guessing rather than successful memory. Low-confidence recognition accuracy also did not differ by age group. Likewise, the difference in encoding judgment reaction time for subsequently remembered items (Hit-HC) and subsequently forgotten items (Miss) did not differ across age groups.

| **Behavioral measure** | **Overall** | **Children** | **Adolescents** | **Young Adults** | **Older Adults** | **F** | **p** |
| --- | --- | --- | --- | --- | --- | --- | --- |
| Hit-HC rate | 0.44 ± 0.14 | 0.37 ± 0.14 | 0.43 ± 0.13 | 0.48 ± 0.11 | 0.48 ± 0.15 | 4.11 | 0.008 |
| Hit-LC rate | 0.11 ± 0.09 | 0.13 ± 0.09 | 0.12 ± 0.09 | 0.11 ± 0.06 | 0.09 ± 0.11 | 0.86 | 0.463 |
| FA-HC rate | 0.17 ± 0.14 | 0.17 ± 0.14 | 0.15 ± 0.12 | 0.11 ± 0.09 | 0.26 ± 0.14 | 6.98 | <0.001 |
| FA-LC rate | 0.10 ± 0.09 | 0.13 ± 0.09 | 0.10 ± 0.08 | 0.11 ± 0.08 | 0.07 ± 0.09 | 2.03 | 0.113 |
| Low-confidence recognition accuracy | 0.01 ± 0.06 | 0.00 ± 0.06 | 0.02 ± 0.05 | 0.01 ± 0.07 | 0.02 ± 0.06 | 0.83 | 0.480 |
| Hit-HC RT – Miss RT (s) | 0.03 ± 0.11 | -0.02 ± 0.12 | 0.02 ± 0.09 | 0.06 ± 0.09 | 0.05 ± 0.14 | 2.51 | 0.064 |

*3.1.2 Subsequent memory performance*


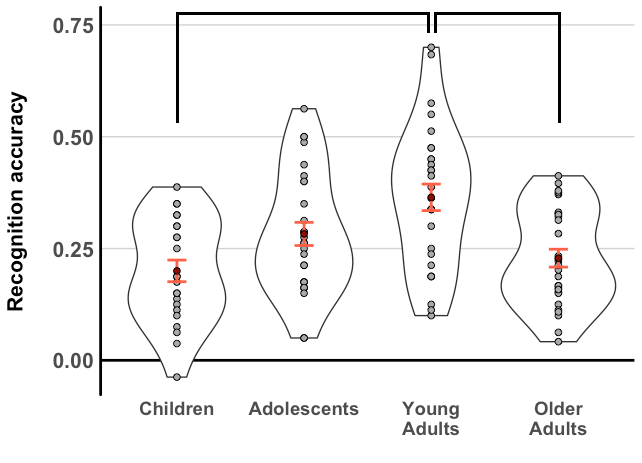


**Supplementary Figure 1.** Effect of age group on recognition accuracy. Violin plots demonstrating greater recognition accuracy in young adults than children and older adults. Recognition accuracy is calculated as the proportion of high-confidence hits minus the proportion of high-confidence false alarms (pHit-HC – pFA-HC). The dark red circles identify age group means, and error bars reflect ±1 standard error.

*3.2 Subsequent memory effect and age*

**Supplementary Table 3. Brain regions where negative subsequent memory effect was observed: activation for subsequently remembered items was lower than activation for subsequently forgotten items.**

| Brain Region | | Voxels | peak T | x (mm) | y (mm) | z (mm) |  |
| --- | --- | --- | --- | --- | --- | --- | --- |
| Right Inferior Parietal | | 1531 | 9.05 | 56 | -54 | 40 |  |
| Right Middle Frontal | | 1576 | 7.62 | 24 | 56 | 22 |  |
| Middle Frontal | |  | 6.34 | 34 | 48 | 0 |  |
| Middle Frontal | |  | 6.00 | 36 | 22 | 40 |  |
| Superior Frontal | |  | 5.67 | 30 | 64 | 4 |  |
| Midcingulate | | 415 | 7.44 | 2 | -18 | 38 |  |
| Posterior Cingulate | | 1008 | 7.03 | 8 | -48 | 28 |  |
| Precuneus | |  | 6.12 | 2 | -66 | 34 |  |
| Posterior Cingulate | |  | 5.73 | -8 | -50 | 30 |  |
| Anterior Cingulate | | 969 | 6.93 | 6 | 42 | 0 |  |
| Anterior Cingulate | |  | 6.15 | 2 | 36 | 18 |  |
| Left Inferior Parietal | | 239 | 6.54 | -60 | -44 | 38 |  |
| Angular Gyrus | |  | 5.38 | -54 | -60 | 40 |  |
| Left Middle Frontal | | 110 | 6.33 | -36 | 42 | 24 |  |
| Right Middle Temporal | | 162 | 6.12 | 62 | -22 | -10 |  |
| Right Superior Frontal | | 90 | 6.01 | 14 | 24 | 54 |  |
| Left Postcentral | | 42 | 5.92 | -60 | -24 | 24 |  |
| Left Superior Frontal | | 13 | 5.29 | -22 | 54 | 20 |  |
| Right Insula | | 16 | 5.28 | 32 | 16 | -14 |  |
| Midcingulate | | 20 | 5.20 | 10 | 34 | 34 |  |
|  | Table shows up to four maxima in a cluster more than 16mm apart, and clusters >10 voxels  Brain region labels identified with aal atlas; coordinates in MNI space | | | | | | |


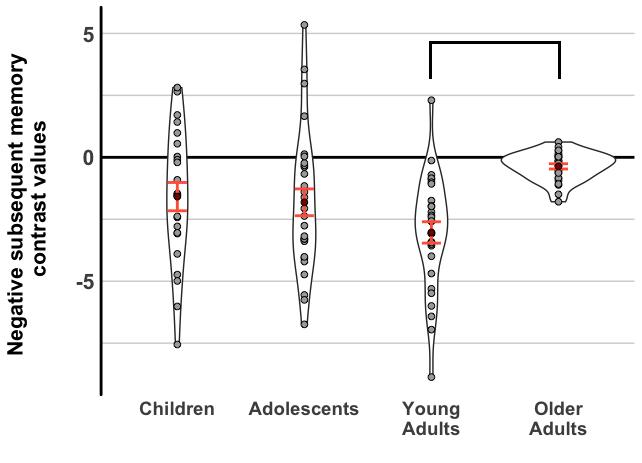

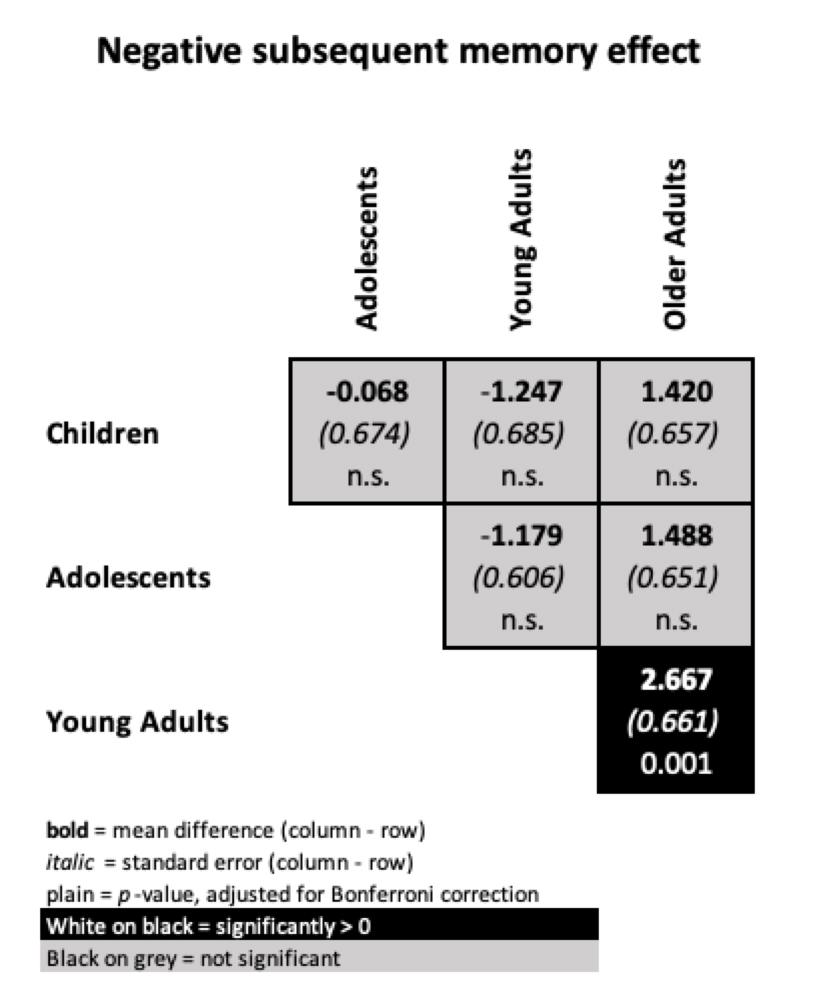


**Supplementary Figure 2: Age group pairwise comparisons for negative subsequent memory effects.** Older adults show reduced negative SME relative to young adults.

**Supplementary Table 4. ANCOVA: Effect of age group on negative subsequent memory effect**

Dependent variable: Negative subsequent memory effect

| Source | Type III Sum of Squares | df | Mean Square | F | Sig. (p-value) | η^2^_P_ |
| --- | --- | --- | --- | --- | --- | --- |
| **Corrected model** | **113.515** | **5** | **22.703** | **4.362** | **0.001** | **0.175** |
| **Intercept** | **59.709** | **1** | **59.709** | **11.472** | **0.001** | **0.100** |
| ***Age group*** | ***85.441*** | ***3*** | ***28.480*** | ***5.472*** | ***0.002*** | ***0.137*** |
| Gender | 6.917 | 1 | 6.917 | 1.329 | 0.252 | 0.013 |
| Avg motion | 2.060 | 1 | 2.060 | 0.396 | 0.531 | 0.004 |
| Error | 536.087 | 103 | 5.205 |  |  |  |
| Total | 966.309 | 109 |  |  |  |  |
| Corrected total | 649.602 | 108 |  |  |  |  |

*r^2^* = 0.175 (adjusted *r^2^* = 0.135), *n* = 109

*Supplementary Results: Positive subsequent memory effect and age*

Positive SME was identified by the contrast of activation to subsequently remembered items (Hit-HC) greater than activation to subsequently forgotten items (Miss). Across all participants positive SME was found in bilateral occipital cortex, bilateral fusiform gyrus/parahippocampal gyrus, and bilateral retrosplenial cortex (Supplementary Figure 3, Supplementary Table 5). Positive SME was extracted, per participant, and the average of contrast values per participant across these regions as a single region of interest were used in further analyses. Positive SME differed by age as evident in a main effect of age group controlling for gender and average framewise displacement, *F*(3,103) = 12.58, *p* < 0.001, *η^2^_P_* = 0.27 (Supplementary Figure 3). Posthoc pairwise comparisons of estimated marginal means revealed older adults demonstrated decreased positive SME relative to children (*p* = 0.045), adolescents (*p* < 0.001), and young adults (*p* < 0.001). Furthermore, children demonstrated decreased positive SME relative to young adults (*p* = 0.014). See Supplementary Figure 4 for statistics regarding pairwise comparisons, and Supplementary Table 6 for the results of the full model. The effect of age on positive SME remains significant after co-varying out recognition accuracy, in addition to gender and average framewise displacement.

**Supplementary Figure 3. Positive subsequent memory effect across and between age groups**. *Left:* Positive SME contrast (high confidence hit > miss, positive values) shows significant clusters in bilateral occipital cortex, bilateral fusiform gyrus/parahippocampal gyrus, and bilateral retrosplenial cortex. We identified regions using a voxelwise significance threshold of *p* < 0.05 FWE-corrected. Significant clusters are displayed on the sample’s averaged T1 structural image; coordinates are provided in MNI space. *Right:* Violin plots demonstrating magnitude of positive SME is reduced in older adults compared to children, adolescents and young adults. There is a significant effect of age group on positive SME, *F*(3,103) = 12.58, *p* < 0.001, *η^2^_P_* = 0.27*.* The dark red circles identify age group means, and error bars reflect ±1 standard error.


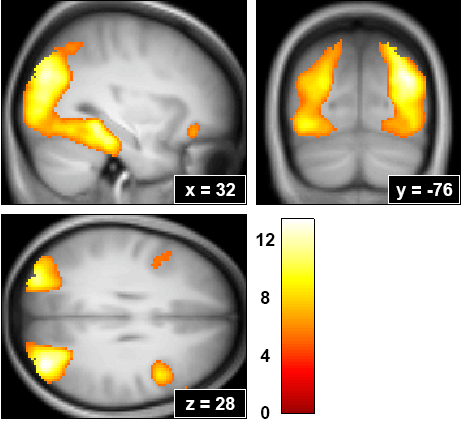

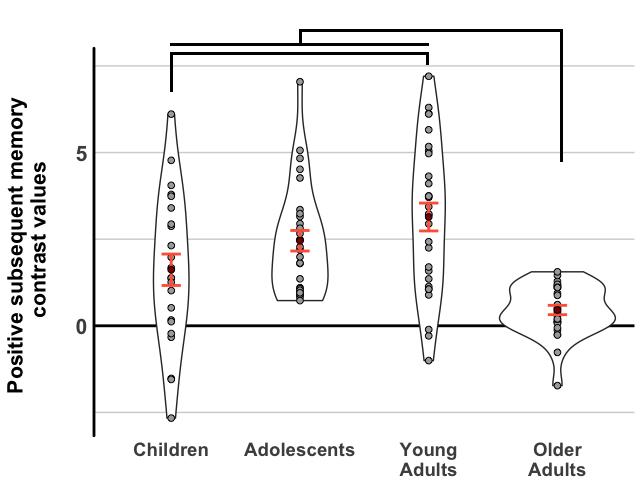


**Supplementary Table 5. Brain regions where positive subsequent memory effect was observed: activation for subsequently remembered items was higher than activation for subsequently forgotten items.**

| Brain Region | | Voxels | peak T | x (mm) | y (mm) | z (mm) |  |
| --- | --- | --- | --- | --- | --- | --- | --- |
| Right Middle Occipital Cortex | | 9770 | 13.43 | 36 | -78 | 28 |  |
| Fusiform Gyrus | |  | 12.10 | 30 | -40 | -12 |  |
| Inferior Temporal | |  | 10.54 | 46 | -62 | -10 |  |
| Middle Occipital | |  | 10.02 | 32 | -84 | 2 |  |
| Left Middle Occipital Cortex, | | 7826 | 12.86 | -28 | -84 | 30 |  |
| Inferior Temporal Lobe, | |  | 11.68 | -44 | -66 | -10 |  |
| Middle Occipital | |  | 10.49 | -30 | -86 | 6 |  |
| Fusiform Gyrus | |  | 10.04 | -30 | -32 | -22 |  |
| Right Inferior Frontal, Operculum | | 395 | 8.85 | 44 | 10 | 28 |  |
| Right Inferior Frontal, Triangular | | 233 | 7.87 | 48 | 36 | 12 |  |
| Inferior Frontal, Triangular | |  | 5.10 | 56 | 26 | 26 |  |
| Right Orbitofrontal Cortex | | 135 | 7.36 | 26 | 32 | -12 |  |
| Left Middle Frontal ,Orbital | | 74 | 6.11 | -34 | 36 | -14 |  |
| Left Inferior Frontal, Triangular | | 78 | 5.60 | -44 | 30 | 14 |  |
| Left Precentral | | 22 | 5.27 | -44 | 8 | 30 |  |
|  | Table shows up to four maxima in a cluster more than 16mm apart, and clusters >10 voxels  Brain region labels identified with aal atlas; coordinates in MNI space | | | | | | |


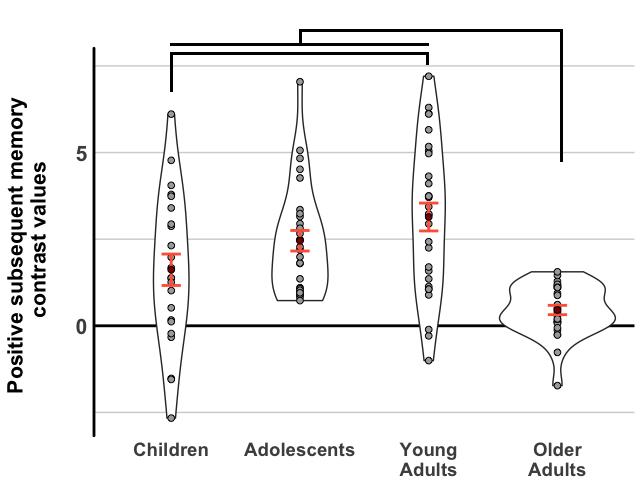

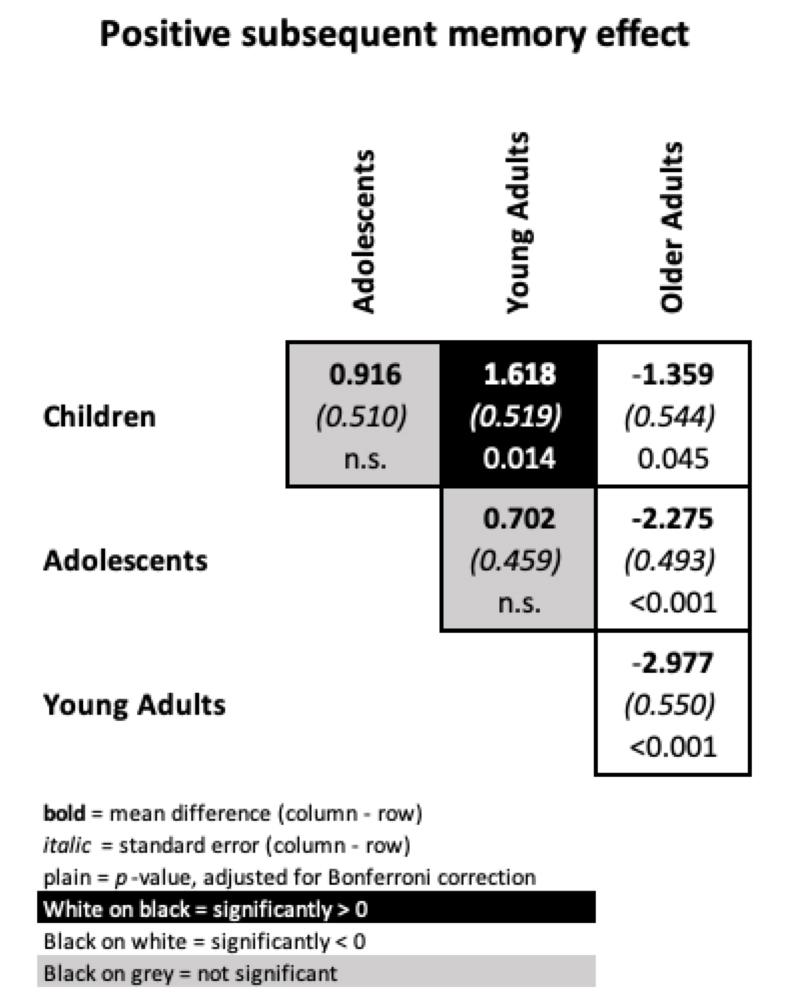


**Supplementary Figure 4: Age group pairwise comparisons for positive subsequent memory effect.** Older adults show reduced positive SME relative to all younger groups. Children also show reduced positive SME relative to young adults.

**Supplementary Table 6. ANCOVA: Effect of age group on positive subsequent memory effect**

Dependent variable: Positive subsequent memory effect

| Source | Type III Sum of Squares | df | Mean Square | F | Sig. (p-value) | η^2^_P_ |
| --- | --- | --- | --- | --- | --- | --- |
| **Corrected model** | **124.748** | **5** | **24.950** | **8.364** | **<0.001** | **0.289** |
| **Intercept** | **37.072** | **1** | **37.072** | **12.427** | **0.001** | **0.108** |
| ***Age group*** | ***112.603*** | ***3*** | ***37.534*** | ***12.583*** | ***<0.001*** | ***0.268*** |
| Gender | 8.349 | 1 | 8.349 | 2.799 | 0.097 | 0.026 |
| Avg motion | 1.572 | 1 | 1.572 | 0.527 | 0.470 | 0.005 |
| Error | 307.254 | 103 | 2.983 |  |  |  |
| Total | 837.293 | 109 |  |  |  |  |
| Corrected total | 432.002 | 108 |  |  |  |  |

*r^2^* = 0.289 (adjusted *r^2^* = 0.254), *n* = 109

*3.3 Subsequent memory effect and recognition accuracy*

**Supplementary Table 7. Two-step linear regression model for negative subsequent memory effect**: Model 1 tests association between negative SME and recognition accuracy. Model 2 adds interaction between negative SME and age group

Dependent variable: Recognition accuracy

| Source | | | | Sum of Squares | | | | | df | | | | | Mean Square | | | F | | | | | Sig. (p-value) | | | | | Adj. R^2^ | |
| --- | --- | --- | --- | --- | --- | --- | --- | --- | --- | --- | --- | --- | --- | --- | --- | --- | --- | --- | --- | --- | --- | --- | --- | --- | --- | --- | --- | --- |
| **Model 1** | | | | **.668** | | | | | **4** | | | | | **0.167** | | | **10.868** | | | | | **<0.001** | | | | | **0.268** | |
| **Model 2 (moderation)** | | | | **.858** | | | | | **5** | | | | | **0.172** | | | **12.556** | | | | | **<0.001** | | | | | **0.349** | |
|  |  | | | | | | |  | | |  | | | | |  | | | |  | | | | | |  | |  |
| Variables (Model 1) | | | *β* | | | | | *t* | | | | Sig. (p-value) | | | | | | | *r_P_* | | | | | |  |  |  |  |
| (Constant) | |  | | | | -1.957 | | | | | | 0.053 | | | | | |  | | | | | |  |  |  |  |  |
| ***NegSME*** | | ***-0.431*** | | | | ***-5.141*** | | | | | | ***<0.001*** | | | | | | ***-0.450*** | | | | | |  |  |  |  |  |
| **Age group** | |  | | | | **2.583** | | | | | | **0.011** | | | | | |  | | | | | |  |  |  |  |  |
| Gender | |  | | | | 0.475 | | | | | | 0.636 | | | | | |  | | | | | |  |  |  |  |  |
| **IQ** | | **0.274** | | | | **3.216** | | | | | | **0.002** | | | | | | **0.294** | | | | | |  |  |  |  |  |
| Added variable (Model 2) | |  | | |  | | | | |  | | | | |  | | | | | |  |  |  |  |  |  |  |  |
| ***NegSME * age group*** | | | ***-0.855*** | | | | ***-3.730*** | | | | | | ***<0.001*** | | | | | | ***-0.345*** | | | |  |  |  |  |  |  |

*Supplementary Results: Positive subsequent memory effect and recognition accuracy*

In testing the association between positive SME and recognition accuracy, we used a regression model predicting recognition accuracy which included positive SME, age group, gender, and IQ. The overall regression model was significant: *F*(4,104) = 5.45, *p* = 0.001, adjusted *R^2^* = 0.14. Positive SME was significantly associated with recognition accuracy after controlling for gender, IQ, and age group: *β* = 0.26, *t* = 2.70, *p* = 0.008, such that stronger positive SME was associated with better recognition accuracy. Results of the full model are reported in Supplementary Table 8 (Model 1).

We further tested whether age group moderated the association between positive SME and recognition accuracy. We did not find evidence of moderation given that the interaction term was not significant (*β* = 0.27, *t* = 1.04, *p* = 0.299) and its addition did not significantly improve the model (ΔR^2^ = 0.009). Statistical values for the interaction term are reported in Supplementary Table 8 (Model 2). Moreover, the moderation by age group on negative subsequent memory/recognition accuracy was significantly greater than the moderation by age group on positive subsequent memory/recognition accuracy (Steiger Z = 1.92, 1-tail *p* = 0.027), highlighting the specificity of the moderation to negative SME. This suggests that the subset of the default mode network which shows negative SME supports memory performance differentially across development and across the lifespan. Comparatively, the occipital, frontal, and medial temporal lobe regions that show positive SME support memory performance more consistently across age groups.

**Supplementary Table 8. Two-step linear regression model for positive subsequent memory effect**: Model 1 tests association between positive SME and recognition accuracy. Model 2 adds interaction between positive SME and age group

Dependent variable: Recognition accuracy

| Source | | Sum of Squares | | | | | | | df | | | | | Mean Square | | | F | | | | | Sig. (p-value) | | | | | Adj. R^2^ | |
| --- | --- | --- | --- | --- | --- | --- | --- | --- | --- | --- | --- | --- | --- | --- | --- | --- | --- | --- | --- | --- | --- | --- | --- | --- | --- | --- | --- | --- |
| **Model 1** | | **.393** | | | | | | | **4** | | | | | **0.098** | | | **5.450** | | | | | **0.001** | | | | | **0.141** | |
| **Model 2 (moderation)** | | **.412** | | | | | | | **5** | | | | | **0.082** | | | **4.581** | | | | | **0.001** | | | | | **0.142** | |
|  |  | | | | | | |  | | |  | | | | |  | | | |  | | | | | |  | |  |
| Variables (Model 1) | | | | *β* | | | | *t* | | | | Sig. (p-value) | | | | | | | *r_P_* | | | | | |  |  |  |  |
| (Constant) | | |  | | | -0.817 | | | | | | 0.416 | | | | | |  | | | | | |  |  |  |  |  |
| ***PosSME*** | | | ***0.255*** | | | ***2.695*** | | | | | | ***0.008*** | | | | | | ***0.255*** | | | | | |  |  |  |  |  |
| Age group | | |  | | | 2.244 | | | | | | 0.027 | | | | | |  | | | | | |  |  |  |  |  |
| Gender | | |  | | | 0.356 | | | | | | 0.723 | | | | | |  | | | | | |  |  |  |  |  |
| **IQ** | | | **0.259** | | | **2.717** | | | | | | **0.008** | | | | | | **0.257** | | | | | |  |  |  |  |  |
| Added variable (Model 2) | | |  | |  | | | | |  | | | | |  | | | | | |  |  |  |  |  |  |  |  |
| *PosSME * age group* | | | | *0.271* | | | *1.043* | | | | | | *0.299* | | | | | | *0.102* | | | |  |  |  |  |  |  |

|  |  |  |  |  |  |  |
| --- | --- | --- | --- | --- | --- | --- |

*Supplementary Results: Regional negative subsequent memory effect*

We performed a series of exploratory analyses using individual regions demonstrating negative subsequent memory effect. That is, rather than combining all regions into a single region of interest, we extracted values for each participant from separate clusters. These analyses were limited to clusters greater than 50 voxels, or the nine largest clusters listed in Supplementary Table 3. As these analyses are exploratory, we set alpha equal to 0.05, and did not correct for the multiple regions being tested.

First, we investigated whether there was an effect of age group in the negative SME of individual regions. Broadly, the pattern of results from the individual regions matches the overall pattern of the combined region of interest; negative SME is generally consistent between children and adolescents, while young adults show the strongest (most negative) negative SME and older adults show the weakest (least negative) (Supplementary Table 9). In all regions except for superior frontal cortex, we found a statistically significant group difference between young adults and older adults (p < 0.05). Furthermore, in the right middle frontal cortex, young adults show a stronger response than children (p = 0.004) and adolescents show a stronger response than older adults (p = 0.005). No other group differences reached statistical significance.

Next, we examined associations between regional negative SME and recognition memory. Negative SME in all regions was significantly associated with recognition memory, with associations ranging in size from small (midcingulate: -0.257) to moderate (right middle frontal cortex: -0.418) (Supplementary Table 10).

Finally, we tested the moderation effect of age group on the association between regional negative SME and recognition accuracy. As in the prior, single-region analysis (Methods 3.5.3: Group Analyses), we used linear regression models that also included age group, gender and IQ. To determine whether age group moderated this association, we repeated the previous linear regression models with the addition of an interaction term of age group and SME. A significant interaction term was interpreted as evidence of moderation of the association between SME and recognition accuracy by age group. Age group significantly moderated the relationship between negative SME and recognition accuracy in all regions (p < 0.05). *T*- and p-values for interaction terms, signifying moderation effect, are reported in Supplementary Table 10.

**Supplementary Table 9. Age group differences in regional negative subsequent memory effect.**

| Brain Region | Children | Adolescents | | Young Adults | Older Adults | *F* | p |  |
| --- | --- | --- | --- | --- | --- | --- | --- | --- |
| Right Inferior Parietal | -2.24 ± 3.83 | -2.69 ± 4.07 | | -4.36 ± 2.70 | -0.59 ± 0.73 | 7.378 | <0.001 |  |
| Right Middle Frontal | -1.60 ± 4.95 | -4.48 ± 6.37 | | -6.02 ± 4.51 | -0.34 ± 0.75 | 9.043 | <0.001 |  |
| Midcingulate | -1.39 ± 2.84 | -1.32 ± 1.96 | | -2.09 ± 2.62 | 0.01 ± 0.68 | 4.778 | 0.004 |  |
| Posterior Cingulate | -1.19 ± 2.71 | -1.89 ± 3.75 | | -2.65 ± 2.99 | -0.09 ± 0.87 | 4.386 | 0.006 |  |
| Anterior Cingulate | -1.51 ± 2.29 | -1.71 ± 2.11 | | -2.24 ± 2.17 | -0.45 ± 1.37 | 4.102 | 0.009 |  |
| Left Inferior Parietal | -1.48 ± 2.66 | -1.76 ± 2.19 | | -2.11 ± 2.51 | -0.44 ± 1.15 | 3.168 | 0.027 |  |
| Left Middle Frontal | -3.21 ± 4.84 | -1.76 ± 5.61 | | -1.76 ± 4.89 | -0.80 ± 1.80 | 3.655 | 0.015 |  |
| Right Middle Temporal | -1.70 ± 3.14 | -1.68 ± 3.02 | | -2.66 ± 2.71 | -0.24 ± 0.84 | 4.393 | 0.006 |  |
| Right Superior Frontal | -2.11 ± 3.45 | -1.45 ± 3.31 | | -2.06 ± 2.52 | -0.49 ± 0.64 | 2.219 | 0.090 |  |
| Brain region labels identified with aal atlas | | |  | | | | | |

**Supplementary Table 10. Regional negative subsequent memory effect: bivariate correlation with recognition accuracy and age group moderation of the association between regional negative subsequent negative memory effect and recognition accuracy.**

|  | Bivariate correlation of regional negSME with recognition accuracy | | | | Moderation effect of age group on negSME – recognition accuracy association | | | |  |
| --- | --- | --- | --- | --- | --- | --- | --- | --- | --- |
| Brain Region | *r* | p | | | *t* | | p | |  |
| Right Inferior Parietal | -0.411 | <0.001 | | | 1.993 | | 0.049 | |  |
| Right Middle Frontal | -0.418 | <0.001 | | | 2.806 | | 0.006 | |  |
| Midcingulate | -0.257 | 0.007 | | | 3.627 | | <0.001 | |  |
| Posterior Cingulate | -0.353 | <0.001 | | | 2.449 | | 0.016 | |  |
| Anterior Cingulate | -0.411 | <0.001 | | | 2.135 | | 0.035 | |  |
| Left Inferior Parietal | -0.311 | 0.001 | | | 3.199 | | 0.002 | |  |
| Left Middle Frontal | -0.267 | 0.005 | | | 2.049 | | 0.043 | |  |
| Right Middle Temporal | -0.359 | <0.001 | | | 3.568 | | 0.001 | |  |
| Right Superior Frontal | -0.307 | 0.001 | | | 2.339 | | 0.021 | |  |
| Brain region labels identified with aal atlas | | |  |  | |  | |  | |
